# Strategies for men’s engagement and its effectiveness in improving child health and immunization—a rapid review

**DOI:** 10.3389/fpubh.2025.1539190

**Published:** 2025-05-26

**Authors:** Sarah Nabia, Myra Betron, Elizabeth Arlotti-Parish, Amanda Varnauskas, Chinelo Cynthia Nduka, Angioha Pius, Jean Munro, Katharine Bagshaw, Chizoba Barbara Wonodi

**Affiliations:** ^1^USAID’s MOMENTUM Country and Global Leadership, International Vaccine Access Center (IVAC), Department of International Health, Bloomberg School of Public Health, Johns Hopkins University, Baltimore, MD, United States; ^2^Department of International Health, Bloomberg School of Public Health, Johns Hopkins University, Baltimore, MD, United States; ^3^Jhpiego, Baltimore, MD, United States; ^4^Department of Emergency Medicine, School of Medicine, Johns Hopkins University, Baltimore, MD, United States; ^5^Department of Community Medicine, Nnamdi Azikiwe University Teaching Hospital, Nnewi, Nigeria; ^6^Direct Consulting and Logistics Limited, Abuja, Nigeria; ^7^Gavi, The Vaccine Alliance Secretariat, Geneva, Switzerland; ^8^Public Health Institute Contractor with the United States Agency for International Development’s Global Health Training, Advisory, and Support Contract (GHTASC), Washington, DC, United States

**Keywords:** child health, immunization, gender, low-and-middle income countries, program implementation, systematic review

## Abstract

**Introduction:**

Despite widespread evidence and recognition that women bear the disproportionate burden of caregiving, there are major gaps in action geared towards equalizing this burden of care between men and women especially in the context of child health and immunization. The goal of this rapid review is to identify and summarize effective and promising strategies for men’s engagement in child health and immunization in low-and-middle-income countries (LMICs) and further categorize the strategies into its potential for gender transformative outcomes.

**Methods:**

We searched PubMed, Embase and CINAHL databases for peer-reviewed literature and identified grey literature sources through key informant interviews. Twenty-seven papers and/or documents were included in the analysis. Data analysis was done through narrative synthesis, and results have been presented using the various levels of the socio-ecological model (SEM).

**Results:**

Majority strategies were at the “intrapersonal” level of SEM and focused on individual education and awareness building through one-on-one or group communication approaches and practical training. Efficacy of these strategies was measured using various indicators such as knowledge and perception levels, paternal-infant attachment, biological marker levels, and paternal behavior. Joint / shared couples’ decision-making was the only gender transformative outcome reported in this review.

**Discussion:**

We found gaps in community and policy level interventions, and provider-side interventions to positively influence men’s engagement in child health and immunization. Moreover, only two studies measured the strategies’ influence on improving immunization outcomes.

**Conclusion:**

It can be concluded there is a significant need for more evidence on gender-transformative approaches in child health and immunization programming in LMICs.

## 1 Introduction

Gender-related barriers for child health and immunization are widely recognized and well documented. Child health and immunization uptake is affected by social norms affecting women’s mobility, low maternal education levels, limited financial capacity among women, harsh treatment from healthcare workers that especially dissuade female caregivers from accessing care, and practical and social challenges for men to actively participate in childcare-related activities (1–3). Decades of research have established that the balance of power and decision-making in households and relationships favors men, giving women limited control over health decisions, and lack of access to resources which has an impact on the quality of health for themselves and their children (4–7). There is now widespread recognition in the form of global guidelines (3), peer-reviewed research and mainstream media that men within their roles in family (fathers and other male household decision-makers) can play a critical role in maternal, newborn and child health (MNCH) and early childhood development (8). It is well documented that burden of childcare falls disproportionately on women (9, 10), limiting their opportunities to improve their own and their family’s health due to missed healthcare appointments for themselves (11), and loss of income from unemployment (12). While gender norms may inhibit men from supporting the use of health services by women and children, men have been less targeted by health care interventions for MNCH and are also much less involved in their children’s care (13), as it is often seen as the women’s responsibility (10).

There is increasing recognition of the need for men’s engagement and gender transformative approaches in MNCH programming. Male/men’s engagement refers to the involvement of men and boys across life phases in family planning, sexual and reproductive health, maternal and child health, and HIV programs as (a) clients/users; (b) supportive partners; and (c) agents of change to improve health and gender equality outcomes, actively address power dynamics, and transform harmful masculinities (14). Engaging men and boys also include broader efforts to promote equality with respect to sexual relations, caregiving, fatherhood, division of labor, and ending gender-based violence (GBV). Gender transformative programming approach is one type among a continuum of other gender programming approaches, defined by the World Health Organization as those ‘that address the causes of gender-based health inequities through approaches that challenge and redress harmful and unequal gender norms, roles, and power relations that privilege men over women’ (15). The other types of approaches in the gender continuum mentioned above are: (a) a gender unequal approach that perpetuates gender inequality by reinforcing unbalanced norms, roles and relations; (b) a gender-blind approach that ignores gender norms, roles and relations and thereby often reinforces gender-based discrimination; (c) a gender-sensitive approach that considers gender norms, roles and relations but does not address inequality generated by unequal norms, roles or relations; and (d) a gender-specific approach that considers women’s and men’s specific needs or roles but does not seek to change these roles.

Despite the plethora of evidence of the gender-related barriers facing women caregivers that impact child immunization uptake (16–18), there is still limited evidence on how men can be effectively engaged to strengthen infant and child immunization uptake and outcomes (5, 8). There has been more robust research on the benefits of men’s engagement within sexual and reproductive health and rights (SRHR) programs, including maternal and newborn health, but little attention and research is available on engaging men as caregivers and the effect it has on child health outcomes, including immunization (19).

The aim of this paper is to synthesize the evidence base on gender transformative interventions, focusing on men’s engagement as caregivers and couple’s joint decision-making, and their proven and potential impact on immunization and child health outcomes in low- and middle-income countries (LMICs). The research questions answered in this paper are:

- What are the effective and promising men’s engagement interventions that can be integrated into child health and immunization programs and services?
- Which among the effective and promising interventions described above can be categorized as gender transformative interventions that can be integrated into child health programs and services?

## 2 Methods

This review adheres to the Cochrane group’s guidelines for rapid review (20). Based on prior experience of conducting a review on ‘male engagement in reproductive health, maternal, newborn, adolescent and child health’, a search strategy of ‘Medical Subject Headings’ terms and Boolean operators was developed for PubMed, Embase, and CINAHL databases. The search concepts included “male engagement” AND “intervention” AND “newborn or child health or immunization” (see Supplementary material; pages 1 and 2 for detailed search strategy). The inclusion and exclusion criteria for the review were pre-determined based on conversations and multiple iterations with subject matter experts and stakeholders in gender, child health and immunization fields. Inclusion and exclusion criteria applied a time filter (studies published between 2010 and August 2023) and language criteria (English and French). The decision to include both languages—expanding beyond traditional English-only searches—was intentional to ensure that relevant research from both Anglophone and Francophone West African contexts was captured, as the literature review informed implementation research conducted in these regions. The pre-specified eligibility criteria were as follows:

### 2.1 Study design

A broad range of study designs such as randomized controlled trial, quasi-experimental studies, cohort studies, case–control studies, qualitative studies were included. We excluded letters to the editor, opinion pieces and reviews.

### 2.2 Population

We included studies that recruited men caregivers or both men and women caregivers as the recipients of the intervention trying to promote engagement for better child health and immunization in LMICs. The minimum criteria were that men should be engaged as active participants along with partners or as agents of change for promoting child health including childhood immunization.

### 2.3 Intervention

We included studies that assessed the efficacy of different types of men’s engagement interventions in child health improvement and immunization uptake. These could be single or combination of strategies such as individual or group communication, couples’ counselling, mass media communication campaign, or structural interventions aimed at reforming regressive norms.

### 2.4 Comparator(s)

We include studies and program assessments that compare men’s engagement interventions in child health and immunization versus those that do not engage men in child health intervention.

### 2.5 Outcome(s)

The outcomes included effectiveness and/or impact of engaging men in child health and immunization improvement and/or outcomes which were gender transformative.

### 2.6 Screening and selection of studies, data extraction and synthesis

Titles and abstract outputs from the databases were screened by 3 reviewers. Those which progressed to full text screening were also screened by 3 reviewers. Bibliography scan of 4 relevant systematic reviews generated by the search was done. No additional relevant studies were identified. Grey literature sources were gathered through a series of key informant interviews.

A data extraction tool was developed in Microsoft Excel. The tool was discussed with subject matter experts who have prior experience conducting similar reviews, and pilot tested with 5 papers. The data was analyzed using the socio-ecological-model (SEM) framework, a deliberate selection as it is the leading framework used for understanding social and behavior change related to gender (16, 21, 22). The SEM model was used to map the different strategies to the various levels of the model, and to demonstrate the complex interplay between individual, relationship, community, and societal factors that affect men’s participation in child health and immunization. The results have been summarized according to the corresponding levels of the SEM model.

### 2.7 Quality appraisal

Considering the objective to rapidly synthesize heterogeneous intervention studies to inform the development of a large implementation trial, a comprehensive quality appraisal approach was employed rather than a detailed risk of bias assessment. This decision reflects the need to evaluate not only internal validity but also broader methodological rigor, reporting standards, and contextual applicability of the included studies. Quality appraisal tools for randomized control trials, qualitative, case–control, and cohort studies by the Critical Appraisals Skill Programme at the Oxford Centre for Triple Value Healthcare Ltd., and tools for cross sectional studies and quasi experimental studies by the Joanna Briggs Institute (JBI) were used to assess the papers for validity of results, interpretation, and local relevance of the results (23, 24). Papers which partially fulfilled the criteria set forth in the quality appraisal tools were included if it had clear objectives, methodology, and relevant results. The detailed quality appraisal is available in the Supplementary materials (pages 2 to 9).

## 3 Results

### 3.1 State of current knowledge on the topic

#### 3.1.1 Peer-reviewed literature

The search yielded 712 records from 3 databases (Embase = 323, CINAHL = 246, PubMed = 143), of which 187 duplicates were removed. Title and abstract screening of 525 unique records were done and 107 studies progressed to full text screening. Twenty-one peer reviewed studies were included in the analysis.

#### 3.1.2 Grey literature

**Figure 1:**
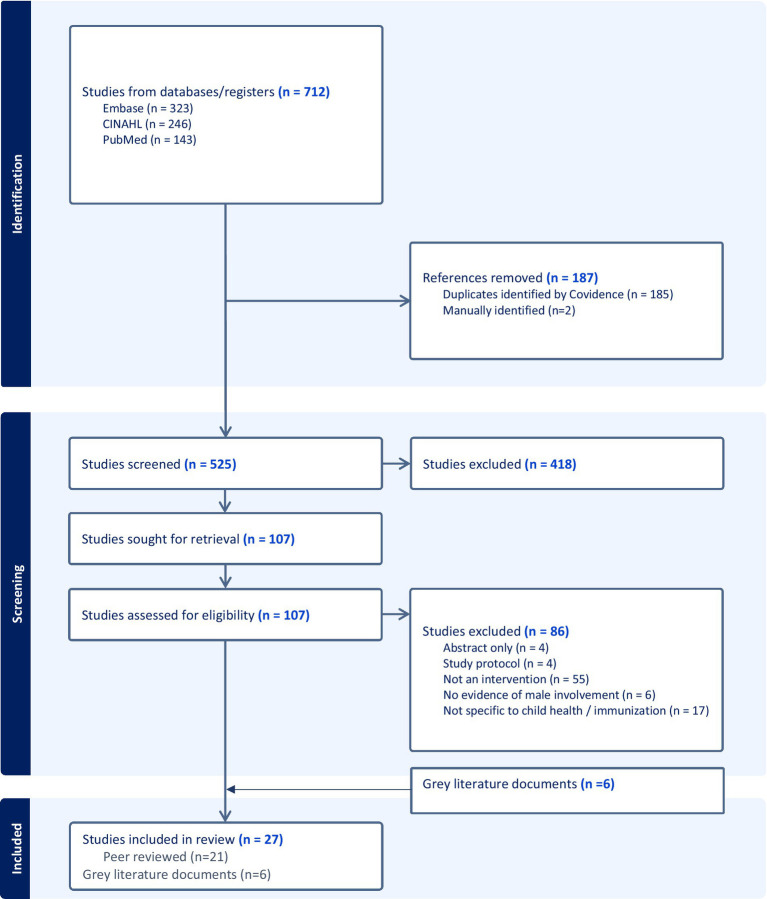
PRISMA diagram.

The key informant interviews yielded 6 studies of optimal quality that were included in the final analysis. (See PRISMA diagram) (Figure 1).

### 3.2 Overview of included studies

**Figure 2:**
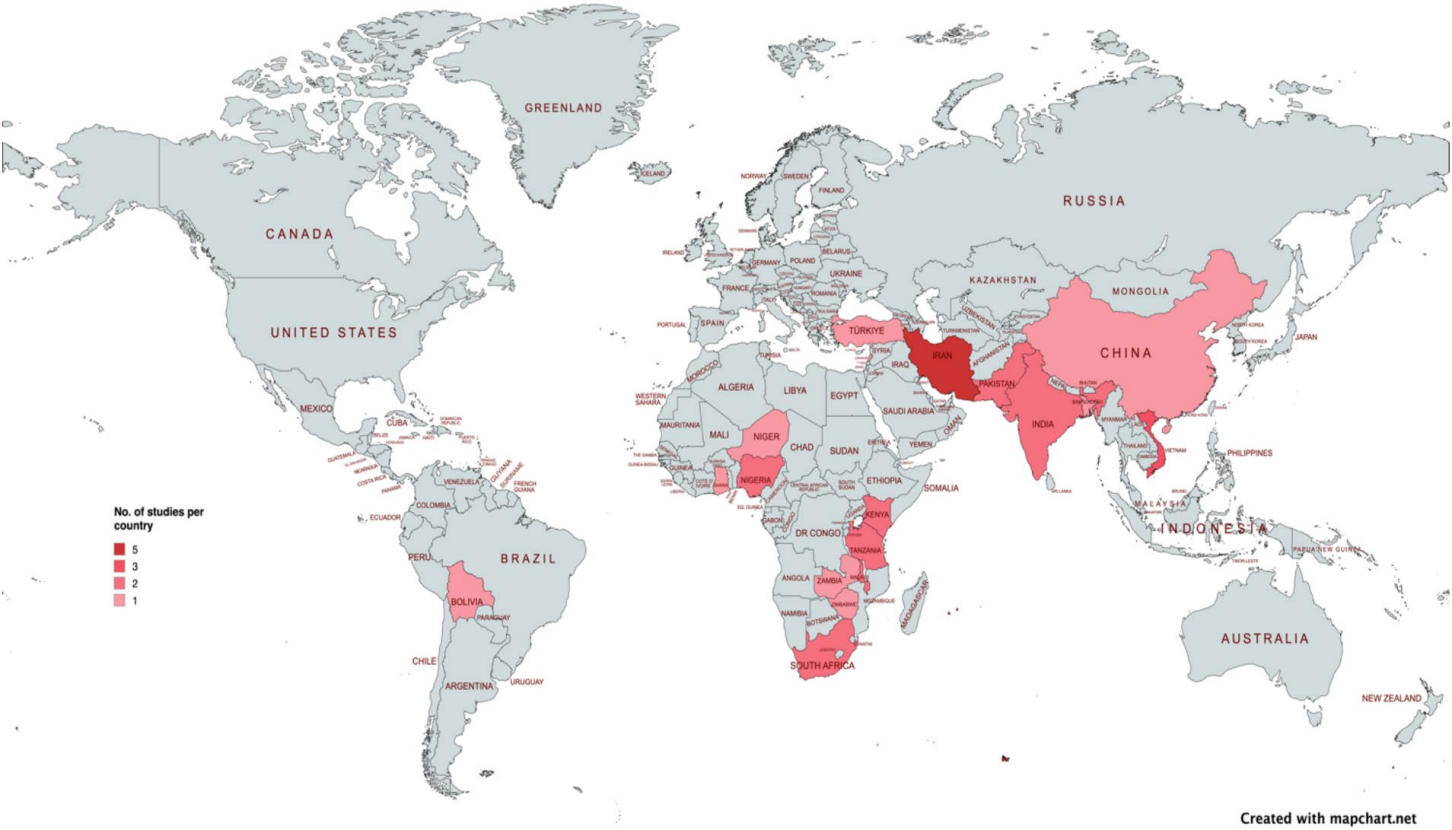
Heat map to show geographical distribution of studies.

The studies are from 18 countries in Asia (n = 8), Africa (n = 9) and South America (n = 1). The geographical distribution of studies is presented in the map (Figure 2).

Eight studies recruited only men and 19 studies (3 of these were case studies within 1 document) recruited both men and women as study participants. A breakdown of the methods can be found in Table 1.

**Table 1:** Overview of evaluation methods.

Evaluation methods	No. of studies	Papers (First author’s last name)	Countries
	Quantitative		
Randomized controlled trial (RCT)	8	Cockcroft, Garcia, Jones, Baheiraei, Rothstein^*^, Alemann (Bolivia), Alemann (Rwanda), Galvin, Yildirim	Nigeria, Kenya, South Africa, Iran, Bolivia, Rwanda, Tanzania, Türkiye
Quasi-experimental study	3	Rahimi, Su, Bich^*^	Iran, China, Vietnam
Longitudinal study with control group	2	Toprak, Sifunda	Türkiye, South Africa
Retrospective cohort study	1	Kalembo	Malawi
Non-randomized impact evaluation study	1	Lyatuu	Tanzania
Pre-post intervention study	3	Maselko, Alemann (Lebanon)^*^	Pakistan, Lebanon
Cross sectional survey	3	Nasreen, Broadbent, Alemann (Ghana and Nigeria)^*^	Bangladesh, Tanzania, Ghana, Nigeria.
	Qualitative		
KIIs, IDIs	7	Mweemba, Fotso, Lusambili, Bich^*^, Rothstein^*^, Alemann (Ghana and Nigeria)^*^, Doughtery, Comrie-Thomson	Zambia, India, Kenya, Tanzania, Bangladesh, Ghana, Nigeria, Niger, Vietnam, Zimbabwe.
FGD	8	Mweemba, Oguntunde, Lusambili, Rothstein, Alemann (Lebanon)^*^, Doughtery, Comrie-Thomson	Zambia, Nigeria, Kenya, Tanzania, Ghana, Haiti, Nigeria, Senegal, Lebanon, Niger, Zimbabwe

* Mixed-methods study design.

The following section summarizes the strategies used to foster and improve men’s engagement in child health across various levels of the socio-ecological model.

### 3.3 Strategies used for men’s engagement in child health and immunization

**Figure 3:**
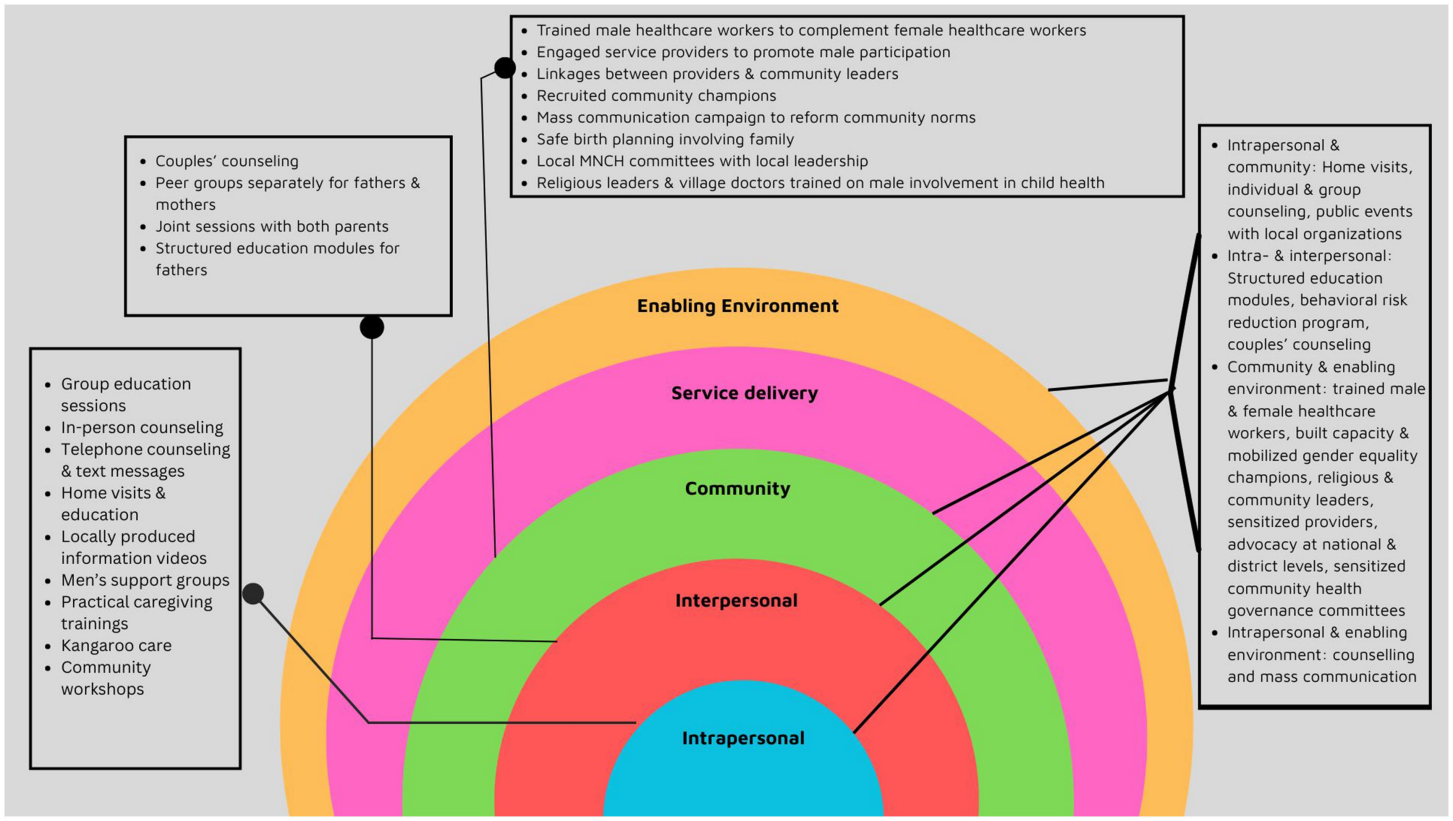
Image showing the SEM levels and interventions.

Table 2 and Figure 3 present a detailed breakdown of the strategies at each level of the SEM. Here we summarize interventions at each level of the SEM by frequency and type.

**Table 2:** Summary of interventions and strategies by SEM levels.

First author, Year	Country	Intervention/strategy	Categories of intervention	Frequency	Intervention/strategy delivered by
SEM level: Intrapersonal					
Alemann 2023 (43)	Bolivia	Community-based group education sessions, with separate content for fathers and for mothers	Group communication	10 sessions for fathers; 9 sessions for mothers	
Alemann 2023 (43)	Lebanon	Group education sessions with fathers/male caregivers and their female partners	Group communication	13 1- to 2-h sessions for male caregivers; women partners joined for 5 sessions	
Baheiraei 2011 (37)	Iran	In-person motivational interviewing/counseling for mothers and telephone-based counseling for both parents on reducing children’s exposure to household smoke	Individual communication	1 in-person and 2 telephone-based sessions for mothers, 3 telephone sessions for fathers	
Cockcroft 2022 (38)	Nigeria	Home visits to pregnant women and spouses. Female and male home visitors interacted with the mother and father, respectively. Conversations included early childhood health, prevention and management of common diseases, routine immunization.	Individual communication	Two-monthly visits during prenatal phase, 1 visit after birth and 1 visit when child is 12–18 months old/	Female and male home visitors
Doughtery 2018 (45)	Niger	Locally produced videos featuring community members focus on key themes, such as dietary diversity, handwashing, exclusive breastfeeding, and complementary feeding practices. Interactive discussions and home visits follow to address any questions.	Individual communication, Group communication		Field mediators/volunteers
Lusambili 2021 (46)	Kenya	Dialogues with men, women and adolescents in community health units	Group communication	Quarterly	Community health volunteers, community health committee members, community health extension workers
Maselko 2019 (54)	Pakistan	Thinking Health Program, a low-intensity psychotherapy program for mothers	Individual communication		Peer counselors
Mweemba 2021 (47)	Zambia	Education films in local language on various health topics	Group communication	4 films screened at each outreach post per month	Ministry of Health
Oguntunde 2019 (39)	Nigeria	Men’s support group for married men. Discussion curriculum included topics child health, immunization, etc.	Group communication		
Rahimi 2022 (42)	Iran	Hands-on training for fathers on neonatal caregiving (feeding, positioning, etc.) in in-patient setting	Practical training	9 sessions over 4 weeks	
Rothstein 2022 (40)	Tanzania	Interpersonal communication, text messages or a combination of both	Individual communication		
Toprak and Erenel 2021 (25)	Türkiye	Kangaroo care by fathers for babies born by cesarean section till baby’s first birthday	Practical training	Twice per week up to 1 year	
Yildirim 2023 (41)	Türkiye	Kangaroo care by fathers for health newborn infants	Practical training	Thrice—4 to 6 h after birth, first day after birth, and third day after hospital discharge	
Gavi 2022 (26)	India	Community workshops with male-specific messaging on overturning norms, infection prevention, spousal communication, etc.	Group communication		
SEM level: Interpersonal					
Kalembo 2013 (33)	Malawi	Couples’ counseling on HIV testing and posttest counseling for those who opted in for HIV testing	Couples’ counseling		
Galvin 2023 (53)	Tanzania	32 peer groups each, separately for fathers and mothers. Peer groups received education either on nutrition only or nutrition and parenting package.	Group education		Community health worker of the same gender as the group
Garcia 2022 (36)	Kenya	Group sessions on responsive parenting. 4 fathers’ only sessions and 12 joint sessions. Intervention arm 1 had group sessions only and intervention arm 2 had 12 group sessions and 4 home visits.	Couple education	16 fortnightly sessions	Community health volunteers
Su and Ouyang 2016 (31)	China	Antenatal breast-feeding education for couples using a ‘father support’ model to foster father’s involvement through physical and emotional aspects	Couple education		
SEM level: Community					
Fotso 2015 (48)	India	Trained male community health workers to complement the work of female health workers	Service provider and stakeholder engagement		
Lyatuu 2018 (34)	Tanzania	Engagement and garnering commitment from service providers, created linkages between service providers and community leaders, recruitment of community champions, integration of male participation initiatives in existing health facility platforms; designed communication campaign for reforming community norms.	community norm building, service provider and stakeholder engagement		
Nasreen 2012 (29)	Bangladesh	Program implemented safe birth planning in the presence of family, recruited MNCH committees with local membership. Religious leaders and village doctors were oriented on male involvement in child health.	service provider and stakeholder engagement		
Multi-levels					
Bich 2016 (30)	Vietnam	Home visits for individual counseling, mass media communication, fathers’ group counseling at monthly antenatal activities, public events in collaboration with farmers’ association	Individual communication, group communication, service provider and stakeholder engagement	Group counseling (monthly)	Village health workers (home visits)
Broadbent 2022 (44)	Tanzania	30-min home visits for counseling and educating mothers, radio and TV spots with taglines.	Individual communication, Mass communication	Radio spots and TV spots were broadcasted 70,000 and 1,198 times respectively; avg. of 3.6 times home visits during study implementation	Community health workers
Jones 2021 (55)	South Africa	Prevention of mother to child standard of care for HIV prevention and ‘protect your family’ module; a manualized, closed, structured behavioral risk-reduction program delivered through a combination of prepartum gender-homogenous group sessions and couples’ counselling, and post-partum individual or couples’ counselling.	individual communication group communication, couples’ counseling	3 prenatal weekly 2-h gender-concordant group sessions and 1 prenatal individual or couples counselling session, along with 2 postpartum individual or couples counselling sessions.	Clinic staff
Sifunda 2019 (32)	South Africa	Prevention of mother to child standard of care for HIV prevention and ‘protect your family’ module; a manualized, closed, structured behavioral risk-reduction program delivered through a combination of prepartum gender-homogenous group sessions and couples’ counselling, and post-partum individual or couples’ counselling.	Individual communication, group communication, couples’ counseling.	3 prenatal weekly 2-h gender-concordant group sessions and 1 prenatal individual or couples counselling session, along with 2 postpartum individual or couples counselling sessions.	Clinic staff
Comrie-Thomson (35)	Bangladesh, Tanzania, Zimbabwe	Training male and female community health workers to engage men in their household visits and counselling, formation, capacity building and mobilization of male groups and gender equality champions/male role models, couples counselling focused on nutrition, etc., mobilization of traditional, religious and other community leaders, community-level campaigns, sensitization of health service providers on gender issues affecting MNCH; advocacy with national and district level authorities on male engagement; sensitization of community health governance committees.	service provider and stakeholder engagement, structural changes		
Alemann 2023 (43)	Ghana, Nigeria	Group education sessions for fathers, socio-behavioral change communication, capacity building of facility-based providers,	Group communication, service providers’ and stakeholder engagement	20 1-h group education sessions for fathers	

#### 3.3.1 Interventions at the intrapersonal level

This is the most basic level of the SEM model and interventions at this level are defined as those that enhance an individual’s knowledge, capacity, agency or resources to take care of their health and well-being. Strategies at the ‘intrapersonal’ level of the SEM synthesized in this analysis can be grouped into 3 types—individual communication, group communication and practical training. These were reported across 14 papers and ranged from home visits by healthcare workers, individual counselling sessions, group sessions, mass media campaigns to practical training on infant care in neonatal intensive care units.

#### 3.3.2 Interventions at the interpersonal level

This is the second level of the SEM, interventions at this level foster interpersonal support and promote health and well-being. The interventions at the ‘interpersonal’ level synthesized in this review have been categorized into two types—namely, couples’ counseling, group education and couples’ education (see Table 2). These were reported in 4 papers included in this analysis.

#### 3.3.3 Interventions at the community level

The ‘community’ level of the SEM includes interventions that promote positive community norms and practices that positively impact the health and well-being of the community. We categorized interventions at this level into two types—service provider and stakeholder engagement and community norm building. Three papers reported ‘community’ level interventions (See Table 2 for details).

#### 3.3.4 Interventions across multiple levels

In addition to the 3 levels mentioned above, the SEM has 2 additional levels—service delivery, and socio-political context levels. In this analysis we did not encounter standalone interventions in the two other levels. Six papers described interventions across multiple levels of the SEM. The combinations of these multiple levels were—individual and interpersonal, community and enabling environment, and individual and community levels (see Table 2).

### 3.4 Outcomes

#### 3.4.1 Overview of outcomes measured

In this review, there were 17 quantitative, 6 qualitative, and 4 mixed methods studies. Only two studies reported immunization outcomes of ‘taking the baby to vaccination’ in a study that tested the implementation of kangaroo care by fathers (25), and age-appropriate compliance of childhood vaccines and knowledge of childhood vaccine timeliness in a study that implemented community workshops with male-specific messaging for fathers, respectively (26). Other quantitative child health outcomes included improvements in child feeding; improvement in child wellness (including lower likelihood of HIV infection, changes in development scores); improved care-seeking (including HIV treatment); and parental knowledge and behavior to promote better child health (uptake of institutional delivery, accompaniment to health facility, etc.). Broader men’s engagement outcomes were also reported, encompassing father’s knowledge on various aspects of child health; joint decision-making and maternal support; parental-child bonding and active parenting.

Eleven qualitative outcomes were reported within the following categories: health behaviors, in the form of facility births; self-reported men’s behaviors (including accompanying women to care visits, participation in MNCH, spousal and child relationships, couple decision-making, men health workers’ engagement with men); men’s knowledge (including understanding of danger signs, MNCH knowledge, different types of knowledge acquired by men through education initiatives); and perception about roles (including perception changes about masculinity and men’s role in fatherhood, and reasons for lack of men’s involvement in child health and care).

It should be noted that some men’s engagement frameworks do not consider increases in men’s knowledge as a relevant outcome (27), despite its consideration as such in the past, given that other factors can contribute to men’s knowledge (such as their literacy levels), and argue that increased knowledge only becomes relevant as it relates to how that knowledge is used, which then falls into other dimensions of men’s engagement. Improvement in knowledge levels is a formative step in changing men’s behavior and while it lies in the causal pathway of gender transformative behavior it cannot be considered one (28). However, given that multiple studies included in this review do include knowledge-related outcomes, they are discussed here as defined by those study authors.

Interventions demonstrating statistically significant findings (in the case of quantitative or mixed methods studies) or otherwise demonstrating positive changes (in the case of qualitative studies) in the above results areas are synthesized in Table 3 by SEM level and type of intervention. The same outcomes are described in more detail following the table.

**Table 3:** Outcomes reported as per the SEM levels.

SEM level and intervention type	Type of outcome associated with the intervention			Gender transformative outcomes
	Health, knowledge and behavioral outcomes			
	Measured child health and related health indicator improvement	Measured change in fathers’ knowledge or behavior	Self-reported change in knowledge or behavior^*^	Couples’ cooperation
SEM level: Community				
Nurturing positive community norms and practices for male involvement	initiation of breast feeding within 1-h of delivery (29); Colostrum feeding (29); couple HIV testing uptake (34)			Joint decision-making (29)
Engaging community stakeholders to promote male involvement in child health	initiation of breast feeding within 1-h of delivery (29); Colostrum feeding (29); couple HIV testing uptake (34)			
SEM level: Interpersonal				
Fostering couple’s communication and cooperation	PMTCT follow up (33), improved child development (36)	increased condom use (33);	Reduction in home delivery and increased hospital delivery (35)	
Couples’ education	improved child development (36); initiation of breast feeding (31), formula feeding rate (31)	infant feeding attitude scale (31)		Joint decision making (36)
SEM level: Individual				
Individual communication	biological markers (37);	father’s knowledge on diarrhea management (38); knowledge on breastfeeding, feeding and micronutrient and hygiene (40); father naming objects for the child, father playing with the child (44)	knowledge of MNCH and family planning among men (46); Men reporting helping with food preparation and feeding, and improved hygiene practices (45); Perceptions about child health and nutrition not being relevant to men, delivery of health and nutrition information skewed towards women (40); accompaniment to facility, etc. (46);	
Group communication	age appropriate compliance of critical vaccines (26)	Father naming objects for the child, fathers playing with their child (44); knowledge of childhood vaccine timeliness (26); handwashing with soap (26)	Men’s perception of male involvement in child health and care (39, 47); Men reporting helping with food preparation and feeding, and improved hygiene practices (45) Men’s perception on masculinity and child care (43); Men’s grasp on danger signs of women and child health (39)	Joint couples’ decision-making (43)
Practical training		Monitoring baby’s development, paternal-infant attachment, taking baby to vaccination, involvement in baby’s care (25); paternal-infant attachment (41, 42)		
SEM level: Multi-level holistic				
Individual, interpersonal, community, and political environment levels (2 or more levels)	HIV infection rate (32); Facility births (35), births attended by skilled health professional (43), initiation of exclusive breast feeding (30, 55); prelacteal feeding (30)	Increased knowledge of MNCH danger signs (43)	Men accompaniment to clinic (35); increased participation in household chores and childcare (43)	Couple communication and decision making on MNCH and household matters (35, 43)

* Self-reported outcomes (from qualitative study designs).

#### 3.4.2 Child health and immunization outcomes

Age-appropriate compliance of childhood vaccines in India was measured in a grey literature source included in this analysis which used program data to assess immunization uptake. It is not clear if a formal evaluation was done. The program implemented community workshop with male-specific messaging for fathers’ help overturn the gender norm that child health is the sole responsibility of the mother, to encourage improved interspousal communication, and to protect their children’s health and as a precursor to their child’s success. An increase in age-appropriate compliance was observed for critical vaccines: increase by 14% for pentavalent; 31.9% for rotavirus; 31% for measles and rubella vaccines (26).

Initiation of breast feeding was measured by Nasreen et al., through a cross-sectional survey in Bangladesh prior which activities were designed to improve men’s engagement in neonatal and child health such as birth planning in the presence of the husband and other family members, and orientation of religious leaders and village doctors to improve men’s participation in childcare activities. Breast feeding was initiated within 1-h of delivery by 65.5% participants in the intervention arm versus 61.3% in the control arm (29). Bich et al. in Viet Nam, and Su and Ouyang in China, also measured initiation of breast feeding in their respective interventions. Bich et al. used a multi-pronged approach of mass media communication, fathers’ group counselling, home visits for individual counselling, and community mobilization activities. The adjusted odds ratio (AOR) of initiating breast feeding in the intervention group was 7.64 compared to those in the control group (30). Su and Ouyang also used education approaches through the ‘father support model’ that imparted education and encouraged greater involvement of fathers to support mothers in feeding practices. Exclusive breast feeding at 6 months was 40% in the intervention arm compared to 17.6% in the control arm where only mothers received this education (31). Prelacteal feeding (AOR: 4.43 in control versus intervention arm), colostrum feeding (95.1% in intervention versus 90.3% in control arm) and formula feeding rate (20% in intervention versus 44.1% in control arm) were other nutrition-related indicators that were measured by Bich et al. (30), Nasreen et al. (29), and Su and Ouyang (31), respectively.

Sifunda et al., conducted a longitudinal study, part of an RCT in Malawi, measuring HIV infection among infants as the outcome. The intervention tested the efficacy of standard “prevention of mother to child transmission” (PMTCT) services plus the ‘protect your family’ program, which included all-mother or all-father groups and couples’ counselling sessions aimed at improving PMTCT uptake. Phase 1 of the study enrolled only mothers and phase 2 enrolled couples. There were increased odds (AOR: 4.55) of infants being infected with HIV in phase 1 (mothers only) compared to phase 2 (couples) of the study (32). Similarly, Kalembo et al., in a retrospective cohort study also measured the association between men’s involvement and completion of PMTCT follow up. Women were encouraged to come to the clinic with their husbands; couples were offered couple counselling and women who came alone received individual counselling. There were increased odds (AOR: 16.8) of completion of follow up among those who engaged as couples in the intervention (33). Even Lyatuu et al. in Tanzania, measured HIV testing uptake as part of PMTCT uptake in a multi-strategy study in six intervention districts that sensitized and engaged clinical and non-clinical staff, employed an integrated approach to improve men’s participation in ANC, created linkages between healthcare providers and community leaders, and recruited community champions to improve male involvement in ANC and PMTCT. The control arm had no intervention. Couples’ HIV testing uptake increased from 11.9 to 36% in the intervention arm as opposed to no significant change in the control districts (34).

Comrie Thompson et al., measured uptake of facility births in a study done in Bangladesh, Zimbabwe and Tanzania which tested multiple strategies across the various SEM levels—household visits and counselling by community health workers, group sessions with men, appointed gender equality champions/male role models, couples counselling on a host of issues including family planning, birth preparedness, etc. sensitization of health providers, sensitization of local religious leaders, and advocacy with government. Through qualitative enquiry, the study found these initiatives were linked to reduction in home births and increase in facility births (35).

Other studies that measured child health outcomes were Garcia et al. in Kenya, a sub-analysis of a cRCT that tested responsive parenting techniques. Arm 1 of the study did educational group sessions only and arm 2 mixed group sessions with home visits, and the control arm had no intervention. In arms 1 and 2, interventions were targeted towards mothers in half the sample and for both parents in other half of the sample. Garcia et al. focused the analysis on arms 1 and 2 of the study which involved fathers. Mothers in villages who participated in the intervention with fathers reported a non-significant trend of increased joint decision-making by the couple versus those where the mother was part of the intervention alone (36). Baheiraei et al. in Iran, an RCT that tested motivational interviewing techniques that included in-person counselling with mothers and telephone counselling with fathers to reduce child’s smoke exposure in households reported infant’s mean urine cotinine concentrations. Infant’s geometric mean urinary cotinine concentrations decreased from 48.72 ng/mg at baseline to 28.68 ng/mg at the 3-month follow-up in the intervention group. In the control group, mean urinary cotinine concentrations decreased from 40.43 to 36.32 ng/mg. The differences between the two groups varying over the course of the 3-month follow-up were statistically significant (*p* = 0.029) (37).

#### 3.4.3 Knowledge and behavioral outcomes

Knowledge about various child health and nutrition issues were measured in Cockcroft et al. and Rothstein et al. In Cockcroft et al., a stepped wedge RCT in Nigeria assessed knowledge on diarrhea and treatment after healthcare workers conducted home visits to all pregnant women and their spouses and counseled them on various safe practices of pregnancy and childcare. Thirty percent more men thought that children with diarrhea should be given extra fluids and 21% more men said they would not give a child medicine to stop diarrhea in the intervention group versus the control group (38). Similarly, Oguntunde et al. in a qualitative study in Nigeria reported men had a better grasp of danger signs for women and child health after the study implemented men’s support groups to raise awareness on women and child health issues (39).

Rothstein et al., a cluster RCT in Tanzania measured knowledge among men on breast feeding, child feeding and micronutrient, and hygiene across 4 study arms that tested communication through either interpersonal communication (IPC), SMS text messages (SMS), combination of both (IPC + SMS), or none of these interventions (control). The breast-feeding knowledge indicator was insignificant, but the child feeding and micronutrient knowledge scores were 0.048 and 0.090 points higher in the IPC and IPC + SMS intervention groups, and the hygiene knowledge score was 0.052 (SMS), 0.065 (IPC), and 0.073 (SMS + IPC) points higher in the intervention arms compared to control arm (40). This study also shed light on the types of knowledge men gained through the interventions such as the importance of seeking antenatal care, and barriers to men gaining knowledge, such as information provision being directed more towards women which made men feel inferior.

Parental-child attachment was measured using different scoring systems in Toprak and Erenel, Yildirim et al. and Rahimi et al. Toprak and Erenel and Yildirim et al. used the postnatal paternal-infant attachment score measured as a composite of three subscales—“patience and tolerance,” “pleasure in interaction” and “affection and pride.” Both studies used kangaroo care techniques and measured father-child attachment. Scores for paternal-infant attachment were higher in the intervention arm (73.53 and 80.57) versus the control arm (70.8 and 56.76) in both studies (25, 41). Rahimi et al. used active parenting techniques and engaged father in-wards to care for their infants. Mean paternal bonding scores were measured using a validated scale originally developed in 1977 that used 10 items to measure maternal-to-infant bonding. As per this scale, paternal-infant bonding improved more in the intervention arm compared to the control arm (42). Alemann et al. in Nigeria and Ghana also qualitatively ascertained improved father-child relationships because of implementation of a multi-pronged approach that included community-based group education session for father, social and behavior change communication, efforts to increase women and girl’s gender equality knowledge, and health system strengthening and capacity building of service providers to do the same (43).

Toprak and Erenal described above also measured other behavioral outcomes such as fathers taking baby to vaccination (60% in intervention versus 17.4% in control arm), fathers monitoring baby’s development (92% in intervention versus 52.2% in control arm), fathers changing baby’s diapers (36% in intervention and 8.6% in control arm), fathers bathing the baby (36% in intervention and 8.6% in control arm) and multiple others (25). Broadbent et al. in Tanzania also measured similar active parenting indicators of ‘likelihood of father naming objects for their child’ and ‘likelihood of fathers playing with their children’. The intervention used radio spots to reach both parents. Fathers who heard radio spots with the intervention messages were 1.42 times more likely to name objects for their child, and 1.58 times more likely to play with their child compared to those who were not exposed to the radio messages (44). Alemann et al. in Nigeria and Ghana qualitatively reported through focus group discussion that men’s participation in MNCH increased (43). The SPRING Project in Niger also showed a positive impact on men’s involvement in nutrition and hygiene, especially increase in percentage of men who supported food preparation and feeding for children after videos featuring local community members promoted high impact maternal, infant, and young child nutrition and hygiene behaviors (45).

Lusambili et al., qualitatively ascertained increase in men’s accompaniment to ANC and under 5 clinics after the study implemented interventions to educate and engage with men through dialogues and group sessions on the importance of men supporting RMNCH services (46). Comrie-Thompson et al. also qualitatively studied in three countries the impact of a range of interventions including training of male and female community health workers to better engage men in their activities, education, behavior change communication and capacity building initiatives for men, mobilizing male role models and traditional, religious and other community leaders, and advocacy with national and district-level authorities. The study found men were more likely to accompany their wives to the clinic in all three countries because of the interventions (35).

Mweemba et al. in Zambia and Alemann et al. in Lebanon reported positive changes in men’s perception of masculinity and how they should engage in child health and care. Both interventions took the couples education approach—Mweemba et al. selected eight educational films produced in the local language that covered a variety of health topics; and Alemann et al. conducted group education sessions with fathers/men and their female partners (43, 47).

#### 3.4.4 Gender-transformative outcomes

Ten of 21 peer-reviewed studies, and 5 of 7 grey literature sources that demonstrated positive changes in health outcomes included interventions that aimed to actively reduce gender inequality between women and men regarding child health decisions and care. However, only 2 peer-reviewed studies and 4 grey literature sources measured gender transformative outcomes. All these peer-reviewed and grey literature studies measured the gender transformative outcomes of joint or shared decision making between couples. Garcia et al. found significant positive associations between men’s engagement (father’s interpersonal support to mother, and shared decision-making) and child development after interpersonal-level responsive parenting and couples communication interventions (36). Nasreen et al. conducted community-level interventions and found non-significant changes in joint decision-making at post-natal care (29). Comrie-Thompson et al. found improved couple communication and decision making related to MNCH and other household matters as a result of the multi-pronged approach that included multi-level holistic interventions including household visits and counselling by community health workers, group sessions, appointed gender equality champions, couples counselling on a host of issues including family planning, birth preparedness, etc. sensitization of health providers, sensitization of local religious leaders, and advocacy with government (35). Alemann et al. also reported improved shared decision making between couples because of community-based group education sessions in Bolivia and Lebanon, in addition to capacity building of community and facility-based providers in Ghana and Nigeria. An overview of these studies can be found in Table 4.

**Table 4:** Studies with gender transformative interventions and outcomes.

Author Country	Intervention with gender transformative elements	Health and non-gender transformative behavioral outcome measured	Gender transformative outcome measured
Peer-reviewed literature			
Nasreen et al. (29) Bangladesh	Community/Nurturing positive community norms and practices for men’s involvement: Implemented safe birth planning in the presence of husband and other family members. Community/Engaging community stakeholders to promote men’s involvement in child health: Recruited MNCH committees in intervention districts consisting of local elites and influencers (e.g., schoolteacher, religious leader, village doctor). As a community norm building activity, imams and village doctors were oriented to improve men’s involvement in child health.	Improved initiation of breastfeeding within 1 h of birth Improved colostrum feeding	Joint decision-making at post-natal care: non-significant results
Garcia et al. (36) Kenya	Interpersonal/couples’ education: Responsive parenting interventions consisted of sessions for mothers, fathers, and couples. Father-only sessions included content on respectful communication between spouses, resolving conflicts, father involvement in childcare and household tasks, and interpersonal support between spouses.	Improved child development Father’s interpersonal support to mother associated with improved child development.	Joint decision making: Mothers who invited fathers to the sessions reported a non-significant trend of increased joint decision-making by the couple vs. those that did not invite fathers.
Su and Ouyang (31) China	Interpersonal/couples’ education: Antenatal breastfeeding education was provided for couples, a “father support” model was used to foster father involvement in decision making with mothers on a feeding model and in supporting breastfeeding practices through emotional and physical aspects.	Increased breastfeeding at 6 months Improved knowledge and attitudes about breastfeeding by fathers	None
Sifunda et al. (32) South Africa	Interpersonal/fostering couples communication and cooperation: Prenatally, participants attended three group intervention (or time-equivalent control) sessions, along with one individual or couples’ session; postnatally, participants attended two individual or couples’ sessions. Both the control and intervention groups received the standard of care (antenatal PMTCT education sessions), but the intervention group also received the ‘Protect Your Family’ intervention. For Phase 1, women participants were recruited without their partners. For Phase 2, both women and men participants were enrolled in the study as couples.	Decreased likelihood of HIV infection among infants	None
Kalembo et al. (33) Malawi	Interpersonal/fostering couples communication and cooperation: Pregnant women were encouraged to come with their partners to the antenatal clinic. Women without partners were counseled individually in private rooms by midwives. Those who came with their partners were counseled together as a couple.	Increased rate of completion of PMTCT follow-up care	None
Lusambili et al. (46) Kenya	Interpersonal/Couples’ education: Community health volunteers did dialogues with men and women quarterly on the barriers to access and use of RMNCH. services, the importance of men supporting women in RMNCH, and the need for men and women to work together to improve health outcomes for their families.	Increased men’s accompaniment to facility-based care visits	None
Lyatuu et al. (34) Tanzania	Community/ Nurturing positive community norms and practices for men’s involvement, Engaging community stakeholders to promote men’s involvement in child health Sensitization, engagement and garnering commitment from health facility staff, Redefining, packaging and delivering generalized key messages to the community focused on encouraging men as partners participation in reproductive and child health services as a whole and framing the messages. this way specifically aimed at addressing barriers related to the pre-conceived notion that ANC clinics are for women only and the stigma associated with HIV-related services.	Increased HIV testing by couples	None
Mweemba et al. (47) Zambia	Individual/group communication: The Ministry of Health selected eight educational films produced in the local language, Bemba, by Medical Aid Films. Each film aimed to provide a factual health education message and covered different health topics.	Increased men’s accompaniment to facility-based care visits	None
Oguntunde et al. (39) Nigeria	Individual/group communication: Men’s support group intervention for married men in their communities. Group activities were designed to improve men’s awareness of maternal and child health needs. The curriculum included discussion of reproductive health, family planning, danger signs in pregnancy, safe delivery, child health, immunization, health related decision making, and basic communication skills.	Increase in men’s knowledge of danger signs for women and children’s health	None
Cockcroft et al. (38) Nigeria	Individual/individual communication: Home visits to pregnant women and their partner, to discuss maternal health risk factors and early childhood health and care, especially prevention and management of diarrhea and routine immunization.	Improved knowledge about diarrhea treatment by fathers	None
Grey literature			
Alemann et al. (43) Nigeria, Ghana	Individual/group communication: Community-based group education sessions for fathers, social and behavior change communication interventions, interventions to increase women and girls’ gender equality knowledge, leadership capacities and networks, and economic capacities and decision-making. Community/engaging community stakeholders to promote male involvement in child health: Health system strengthening and building the capacity of community and facility-based providers.	Increased knowledge of MNCH danger signs, increased births attended by skilled health professionals, increased participation in childcare	Joint couples’ decision making
Alemann et al. (43) Bolivia	Individual/group communication: Community based group education sessions with separate content for fathers and mothers on equitable caregiving and domestic work, and positive parenting.	None	Joint couples’ decision making
Alemann et al. (43) Lebanon	Individual/group communication: Group education sessions with fathers/ male caregivers and their partners on fatherhood, caregiving, violence prevention.	Men’s perception on masculinity and childcare	Joint couples’ decision-making
Gavi et al. (26) India	Individual/group communication: Community workshops for fathers with male specific messaging to help overturn the gender norm that child health is the sole responsibility of the mother, and to encourage improved interspousal communication.	Increase age-appropriate compliance of childhood vaccines, improved hand washing with soap practices	None
Comrie-Thomson et al. (35) Bangladesh Zimbabwe Tanzania	Individual/group communication: Men-only education and behavior change communication group sessions Interpersonal/couples’ education: couples’ counselling Community/engaging community stakeholders to promote male involvement in child health: mobilization of male groups and gender equality champions for peer-to-peer outreach, mobilization of traditional, religious and community leaders, sensitization of health service providers on gender issues affecting MNCH, CHW training on MNCH and male engagement. Socio-political/policy and practice changes: gender and MNCH issues integrated into health service providers’ technical training, infrastructure development support, strengthening emergency transportation and referral systems for men and women, advocacy with national and district level government health departments, sensitization of community health governance committees on gender issues and male engagement.	Increased health facility births	Couples’ communication and decision making

### 3.5 Prospect of intervention effectiveness

We categorized interventions by its potential of being effective, promising, conflicting evidence and no evidence. Effective interventions are defined as those which were rigorously designed and showed positive outcomes. Promising interventions are defined as those which were studied using study designs where rigor and generalizability can be further improved but still showed positive results. Interventions categorized as conflicting, or no evidence did not provide full proof on its effectiveness owing to unclear study rigor (see Table 5).

**Table 5:** Classification of effective, promising, conflicting and no evidence interventions.

Classification	Intervention type by type of outcome(s)			
	Interventions measuring child health and related health outcomes	Interventions measuring knowledge outcomes	Interventions measuring behavioral outcomes	Interventions measuring gender transformative outcomes
Effective if designed well (based on rigorous impact evaluation), i.e., randomized controlled trial, quasi-experimental design, baseline vs. endline data with control group—of the intervention	Individual counselling, couples’ counselling, group sessions exclusive for fathers and mothers on PMTCT (32, 55) Breast feeding education for fathers (31). Motivational interviewing to reduce infant smoke exposure (37). Mass media communication + individual counselling + public events (30) Sensitization and involvement of healthcare staff+ engage community champions + integration of male partner participation in existing ANC health session + redefining public communication strategy (34) Safe birth planning with partner and family + recruited local MNCH committees with community influencers + sensitization of local leaders (29)	Home visits by men HCW to counsel fathers, female HCW to counsel mothers on child health (38) Safe birth planning with partner and family + local MNCH committees + sensitization of local leaders (29)	Practical training offered in the NICU on neonatal care (42) Kangaroo care (25, 41) Breast feeding education for fathers (31)	Community-based group education sessions, with separate content for fathers and for mothers (43) Group education sessions for fathers and couples on responsive parenting (36)
Promising -based on pre-post data but no control group, process evaluation, qualitative evaluation, perspectives and experiences of the expert key informants; more research needed.	Community workshop with male-specific messaging (26)	Men’s support groups for married men which included a MNCH curriculum (39) Quarterly dialogues with men, women, adolescents on RMNCH (46)	Education and communication through radio broadcast + CHW home visits for child health education for mothers (44) Individual counseling for women/couples’ counselling on HIV prevention (33) Educational films on health education (45, 47) Community workshop with male-specific messaging (26) Quarterly dialogues with men, women, adolescents on RMNCH (46)	Fathers’ clubs to transform behavior towards women and children (39) Group education for fathers + capacity building for providers + socio-behavioral change communication intervention + women upliftment strategies (43) Group education for male caregivers and their partners (43) Group education for men + couples’ counselling + engagement with community stakeholders + policy and practice changes (35)
Conflicting evidence			Inter-personal communication + SMS on MNCH best practices (40)	
Not effective, or unclear—based on evaluation or perspectives of the experts.	Low-intensity psychotherapy program for mothers (54)		Home-based outreach by same-gender HCW for fathers and mothers for education (48, 53)	

## 4 Discussion

This review found 21 peer-reviewed implementation research studies and 6 grey literature reports from 2010 to 2024 in LMICs that report child health outcomes. Ten approaches were assessed as ‘effective for child health outcomes if designed well’ and another nine interventions were assessed to be promising for the same (Table 5). Six studies measured child health outcomes such as initiation of breast feeding, colostrum feeding, formula feeding, HIV infection rate, child’s development, and biological markers (26, 29, 31, 32, 36, 37); and three others measured related health indicators such as PMTCT uptake and couples’ HIV testing uptake that could affect a child’s health (33, 34, 43). Three studies measured paternal-infant attachment using different scales of measurement (25, 41, 42). Only six studies included in the review reported gender transformative outcomes, and all six reported the same indicator—joint or shared couples’ decision making (29, 35, 36, 43).

Interventions identified in this review were heavily focused on bringing about knowledge and behavioral change through individual and group education and communication. The interventions assessed as effective were largely standalone or combination of individual and group education efforts with education material tailored to suit context and group composition, couples’ counselling, mass media communication, practical training for fathers on infant caregiving, and promotion of kangaroo care practice by fathers. An intervention of male healthcare workers interacting with fathers and female healthcare workers interacting with mothers in their homes although assessed as effective with significant impact in one study (38), was assessed as not effective or unclear in another study (48); this could be attributed to the lack of rigor in the latter study. Twelve interventions were deemed ‘promising’ in their capability of positively enhancing men’s engagement in child health (Table 5). Promising interventions were standalone or combination of community sensitization techniques such as recruitment of local MNCH committees among village influencers and recruitment of local champions, sensitization of healthcare providers, individual and group health education sessions for parents, use of mass media for health awareness dissemination, and creation of peer support groups (fathers’ clubs) to foster behavior change through education and knowledge sharing (Table 5).

What we did not find is perhaps more telling of the gaps than what we did find. We set out to synthesize evidence on the impact of men’s engagement on child health and immunization but found only two studies that measured immunization-related outcome; “age-appropriate uptake of childhood vaccines” (26) and “taking baby to vaccination” (25), the latter being a secondary outcome in the program. We also sought to identify interventions that engaged men in child health through a gender transformative approach, i.e., changing gender norms and roles, but we found only two studies in the peer-reviewed literature (29, 36), and four in the grey literature (35, 43). We can conclude there is significant dearth of evidence about the association between men’s engagement in childcare and immunization uptake, as well as on the association and pathways on engaging men to achieve gender transformative outcomes and improved child health. These gaps warrant targeted research studies to clearly establish the link between gender sensitive and transformative approaches and improved immunization uptake, with a focus on measuring gender transformative outcome indicators such as improved shared decision making, shift in gender norms of care giving, and changes in gender-based violence related to childcare stress in addition to immunization outcomes, to name a few. While new studies exclusively designed to assess the relationship between gender transformative approaches and immunization uptake may not always be feasible, there needs to be a conscientious effort to integrate immunization uptake monitoring indicators into studies assessing associations between gender-based interventions and child health or study the impact on immunization by including gender transformative approaches into the study design of existing implementation research studies in immunization.

Second, of the interventions reviewed, few gender-transformative approaches have involved engaging men as caregivers through addressing provider-side factors such as health facility policy, health provider influence, training of health workers, and attitudinal change of health workers specifically. Most interventions engaged men through community-level or household interventions.

Third, there was very limited evidence of interventions focusing on transforming community norms on the role of fathers and male caregivers in childcare and its importance in improving child health outcomes, or of interventions focusing on improving the attitudes and approaches of healthcare providers to encourage men’s engagement in child health and care provision. The interventions uncovered in this review are in stark contrast to interventions documented in the behavioral literature from high-income countries (HICs) which largely aim to overcome structural barriers in men’s engagement in child health. Structural interventions seen in HICs range from focusing care systems in policies on the most marginalized people in society, advocating for cultivating a culture of care in workplaces, rethinking and overhauling the way boys are educated about care and their role in it, and normalizing equal parental leave for women and men caregivers among others (49). For example, the Global Boyhood Initiative being implemented in several countries is aiming to study the inequitable gender norms that start early in childhood between ages 4 to 13 years and use the insight to change how boys are taught about care responsibilities and to value hands-on-care and their role in it (50). Equalization of parental leave policy benefits has been a bedrock in the movement towards involving men and normalizing their roles in caregiving. In Poland, the country with one of the world’s most liberal parental leave policies, as a recent result of advocacy and non-governmental effort, the leave policy was further revised to guarantee fathers access to the entire duration of parental leave and financial reimbursement for part of their extended leave (49). However, it is not that structural interventions have not made headway in LMICs. In Eswatini, a mass communication campaign ‘the Babe Locofto campaign’ or the ‘The Good Dad campaign’ since 2019 has reached 230,000 people to celebrate and encourage how fathers can be present and positively influence their children’s lives. The campaign roped in support from prominent champions such as artists, influencers and political figures to popularize it (49).

Although we are yet to see a wave of structural interventions targeting policy change in LMICs, there are traces of efforts attempting to address community values and norms on masculinity and fatherhood. It is also worth reflecting on, and assessing the nature of policies that should be targeted for revision and action in LMICs as the social context greatly varies from that in HICs. For example, the majority of workforce in LMICs are engaged in the informal employment sector, and policies such as parental leave have little to no effect on this workforce. In some LMICs, social norms around family support for care-giving and different living arrangements, such as multi-generational households, highlight the need for care-giving support beyond leave policies. Policy efforts should also focus on shifting core belief systems that place a disproportionate burden of domestic duties on women. Undoubtedly, there is a huge need for policy reforms on this topic in LMICs, but the type of policy that needs to change in these settings warrants more context specific research rather than just following the template used in HICs.

Another comparable area of research to this review is how men engagement strategies and gender transformative approaches have been used in SRHR interventions. The volume of research on the topic of men’s engagement and SRHR is multiple times larger compared to the topic men’s engagement and child health and immunization. McAteer et al., a review of systematic reviews on the former topic included as many as 39 studies in its analysis, of which nearly 40% of the reviews ported a “positive result” on the association between men’s engagement strategies and improvement in SRHR indicators (19). Community mobilization and education techniques have been the more common types of gender transformative strategies used in SRHR programs. Like strategies found in this review, education of men and women either in same-sex or mixed-sex groups has been more commonly delivered compared to others. Very few studies have expanded their scope to address unequal gender dynamics at the structural level, especially in policy. It also highlighted the frequent use of multi-component, multi-level programming approaches with both men and women. There are similarities in this approach with men engagement in child health strategies as we observed multiple studies had multi-component intervention packages ranging from one-on-one education, couples counselling and group education in same-sex or mixed-sex groups. Like the dearth in evidence about the association between men’s engagement and immunization uptake, studies about the direct association between men’s engagement and product coverage or access and disease incidence seem to be an under-studied area in the SRHR space. Like seen in this review, most studies in the SRHR space also measure behavioral and attitudinal change outcomes.

There are still many gaps in the research to understand the effectiveness of interventions seeking to engage men to facilitate and improve child health and immunization outcomes, whether gender transformative or not. Beyond effectiveness, this review found little research or analysis to understand the social processes, challenges, and by-products of engaging men around child health, including filling in the gaps with respect to the following questions: What are the emotional, physical and financial barriers to men’s participation in caregiving? What agency do women have in caretaking? How does men’s involvement in caregiving impact women’s agency in family health? How does men’s participation in caregiving and family health promote more gender equitable norms and relationships?

This review is not without its drawbacks. Due to the linguistic limitations of our team, we restricted including articles only to English and French. This may have caused some bias towards research published by Anglophone and Francophone research groups and universities. We had also not included child mistreatment/abuse indicators in our data abstraction plan since these are not considered health outcomes per se. This limited our capacity to study any association between men’s engagement in childcare and changes in child mistreatment and abuse. In LMICs, there is also a growing body of evidence that engaging men through gender transformative positive parenting programs can reduce intimate partner violence and violence against children and promote gender equality in households, both public health issues (51–53). Future reviews or iterations on this topic should explore additional association with these changes and child health outcomes.

## 5 Conclusion

This review provides future direction for research on the topic of men’s engagement in child health and immunization. First, the sheer volume of research needs to increase to make credible conclusions about effective or not effective strategies on this topic. Second, the existing research in LMICs has until now mainly focused on individual and inter-personal interventions on the consumer or user end. We need to test more strategies at the community level and at the structural or policy level. Third, interventions focusing on the “provider” end are grossly missing in the literature. Future research can explore strategies to train and reform norms among service providers and provider-related policies to enhance men’s engagement in this space. Finally, the current state of evidence on this provides limited basis for mainstreaming gender-transformative considerations into immunization-related programming and rather, calls for further investments in research on the association between men’s engagement and immunization uptake for children (Figure 3).

## Data Availability

The original contributions presented in the study are included in the article/Supplementary material, further inquiries can be directed to the corresponding author.
