# Supplementary material for: Strategies for men’s engagement and its effectiveness in improving child health and immunization—a rapid review
Source: Front Public Health. 2025 May 26;13:1539190. doi: 10.3389/fpubh.2025.1539190 (PMC12146272; doi:10.3389/fpubh.2025.1539190)
Supplement: Supplementary file 1 [file Data_Sheet_1.docx]

Supplementary Material

Strategies for men’s engagement and its effectiveness in improving child health and immunization – A rapid review

Sarah Nabia, Myra Betron, Elizabeth Arlotti-Parish, Amanda Varnauskas, Chinelo Cynthia Nduka, Angioha Pius, Jean Munro, Katharine Bagshaw, Chizoba Barbara Wonodi^*^

*** Correspondence:** Chizoba Barbara Wonodi: cwonodi1@jhu.edu

# Search Strategy

"Father-Child relations"[Mesh] OR "Paternal Behavior"[Mesh] OR "Male engagement"[tiab:~3] OR "Male involvement"[tiab:~3] OR "men’s involvement"[tiab] OR "men’s engagement"[tiab] OR "male influence" [tiab:~3] OR "partner involvement"[tiab:~3] OR "partner engagement"[tiab:~3] OR "partner's involvement"[tiab:~3] OR "partner's engagement"[tiab:~3] OR "paternal involvement"[tiab] OR "partner influence"[tiab:~3] OR "paternal engagement"[tiab:~3] OR "father’s role"[tiab:~3] OR "husband’s role"[tiab:~3] OR "husband’s engagement" [tiab] OR " husband’s involvement" [tiab] OR "husband influence"[tiab:~3] OR "father-child relation*"[tiab] OR "father child relation*"[tiab] OR "masculinities"[tiab] OR "masculinity"[tiab] OR "couple decision making"[tiab] or "spouse decision making" [tiab] OR "equitable decision making"[tiab] OR "joint decision making"[tiab] OR "couple communication"[tiab:~3] OR "couple's communication"[tiab:~3] OR "couple decision-making"[tiab:~3] OR "couple's decision-making"[tiab:~3] OR "equitable decision-making"[tiab:~3] OR "joint decision-making"[tiab:~3]

AND

"Program Evaluation"[Mesh] OR “Program Evaluation*”[tiab] OR “Program Sustainability”[tiab] OR “Program Effectiveness”[tiab] OR “Program Appropriateness”[tiab] OR “program acceptability”[tiab] OR “program cost effectiveness”[tiab] OR “program efficacy”[tiab] OR “program feasibility”[tiab] OR “program impact”[tiab] OR “Intervention”[tiab] OR “clinical intervention”[tiab] OR “program*”[tiab] OR “evaluation”[tiab] OR “economic empowerment”[tiab]

AND

"Infant Health"[Mesh] OR "Infant Care"[Mesh] OR "Neonatal Screening"[Mesh] OR "Intensive Care, Neonatal"[Mesh] OR "Child Health"[Mesh] OR "Vaccination"[Mesh] OR "Immunization"[Mesh] OR "Child Mortality"[Mesh] OR “newborn health”[tiab] OR “child health”[tiab] OR “immuniz*”[tiab] OR “immunis*” [tiab] OR “vaccinat*”[tiab] OR “couples counseling”[tiab] OR “couples counselling” [tiab] OR “early childhood development” [tiab] OR “nurturing care”[tiab] OR “malaria” [tiab]

# Supplementary Tables

Quality Appraisal of included papers

## Qualitative Studies

| First Author’s last name (year of publication) | 1.Was there a clear statement of the aims ofthe research? | 2.Is a qualitative methodology appropriate? | 3. Was the research design appropriate to address the aims of the research? | 4.Was the recruitment strategy appropriate to the aims of the research? | 5. Was the data collected in a way that addressed the research issue? | 6. Has the relationship between researcher and participants been adequately considered? | 7. Have ethical issues been taken into consideration? | 8. Was the data analysis sufficiently rigorous? | 9. Is there a clear statement of findings? | 10. How valuable is the research? |
| --- | --- | --- | --- | --- | --- | --- | --- | --- | --- | --- |
| Fotso (2015) | Yes | Yes | Yes | Yes | Yes | Can't tell | Yes | Yes | Yes | Valuable |
| Oguntunde (2019) | Yes | Yes | Yes | Yes | Yes | No | No | Can't tell | Yes | Valuable |
| Lusambili (2021) | Yes | Yes | Yes | Yes | Yes | Can't tell | Yes | Yes | Yes | Valuable |
| Maselko (2020) | Yes | Yes | Yes | Yes | Yes | Yes | Can't tell | Can't tell | Yes | Valuable |
| Mweemba (2020) | Yes | Yes | Yes | Yes | Yes | Can't tell | No | Yes | Yes | Valuable |
| Alemann (2023) | Yes | Yes | Yes | Can't tell | Yes | Can't tell | Yes | Yes | Yes | Valuable |
| Comrie-Thompson (2015) | Yes | Yes | Yes | Yes | Yes | Can't tell | Yes | Yes | Yes | Valuable |
| Doughtery (2017) | Yes | Yes | Yes | Yes | Yes | Can't tell | Yes | Yes | Yes | Valuable |

## Case-control Studies

| First author’s last name (year) | Section A: Are the results of the trial valid? |  |  |  |  |  |  | Section B: What are the results? |  |  | Section C: Will the results help locally? |  |
| --- | --- | --- | --- | --- | --- | --- | --- | --- | --- | --- | --- | --- |
|  | 1. Did the study address a clearly focused issue? | 2. Did the authors use an appropriate method to answer their question? | 3. Were the cases recruited in an acceptable way? | 4. Were the controls selected in an acceptable way? | 5. Was the exposure accurately measured to minimize bias? | 6 (a). Aside from the experimental exposure, were the groups treated equally? | 6. (b) Have the authors taken account of the potential confounding factors in the design and/or in their analysis? | 7. How large was the treatment effect? | 8. How precise was the estimate of the treatment effect? | 9. Do you believe the results? | 10. Can the results be applied to the local population? | 11. Do the results of this study fit with other available evidence? |
| Toprak (2020) | Yes | Yes | Yes | Yes | Yes | Yes | No | 2.25 | p<0.05 | Yes | Yes | Yes |

## Cohort Studies

| First author’s last name (year) | Section A: Are the results of the study valid? |  |  |  |  |  |  |  | Section B: What are the results? |  |  | Section C: Will the results help locally? |  |  |
| --- | --- | --- | --- | --- | --- | --- | --- | --- | --- | --- | --- | --- | --- | --- |
|  | 1. Did the study address a clearly focused issue? | 2. Was the cohort recruited in an acceptable way? | 3. Was the exposure accurately measured to minimise bias? | 4. Was the outcome accurately measured to minimise bias? | 5. (a) Have the authors identified all important confounding factors? | 5. (b) Have they taken account of the confounding factors in the design and/or analysis? | 6. (a) Was the follow up of subjects complete enough? | 6. (b) Was the follow up of subjects long enough? | 7. What are the results of this study? | 8. How precise are the results? | 9. Do you believe the results? | 10. Can the results be applied to the local population? | 11. Do the results of this study fit with other available evidence? | 12. What are the implications of this study for practice? |
| Kalembo (2013) | Yes | Yes | yes | Yes | Not sure | Yes | Yes | Yes | In univariate analysis, male partner involvement was significantly associated with condom use (OR = 8.3, 95%CI: 3.5–19.6, P,0.001), artificial infant feeding (OR = 3.1, 95%CI: 1.6–6.2, P,0.01), hospital delivery (OR = 29.4, CI: 12.3–70.1, P,0.001)and completion of follow-up in the program (OR = 19.0, CI: 10.1– 35.7, P,0.001) | Very precise | Yes | Yes | Yes | The finding highlights the need for a further study to establish the reasons of LTFU among PMTCT clients at Mwanza. It also calls for PMTCT providers to find ways of tracking clients who are lost to follow-up. |
| Broadbent | Yes | Not clear | Not clear | Yes | Not sure | Yes | Yes | Yes | Fathers showed varying degrees of involvement. About 18% and 23% of fathers were temporarily non-resident at 3 months and 12 months, respectively. The majority of fathers reported that they often help with taking care of the child (70% at both 3 and 12 months). However, only 11% at 3 months and 23% at 12 months mentioned that the baby likes to be held by the father | Moderately precise | Yes | Yes | Yes | child development, and perhaps maternal mental health, programs are likely to benefit from engaging fathers in order to maximize potential impact on the child. |

## Randomized controlled trials

| First author’s last name (year) | Section A: Is the basic study design valid for a randomised controlled trial? |  |  | Section B: Was the study methodologically sound? |  |  |  |  | Section C: What are the results? |  |  | Section D: Will the results help locally? |  |
| --- | --- | --- | --- | --- | --- | --- | --- | --- | --- | --- | --- | --- | --- |
|  | 1. Did the study address a clearly focused research question? | 2. Was the assignment of participants to interventions randomised? | 3. Were all participants who entered the study accounted for at its conclusion? | 4 (a) Were the participants ‘blind’ to intervention they were given? | 4 (b) Were the investigators ‘blind’ to the intervention they were giving to participants? | 4 (c) Were the people assessing/analysing outcome/s ‘blinded’? | 5. Were the study groups similar at the start of the randomised controlled trial? | 6. Apart from the experimental intervention, did each study group receive the same level of care (that is, were they treated equally)? | 7. Were the effects of intervention reported comprehensively? | 8. Was the precision of the estimate of the intervention or treatment effect reported? | 9. Do the benefits of the experimental intervention outweigh the harms and costs? | 10. Can the results be applied to your local population/in your context? | 11. Would the experimental intervention provide greater value to the people in your care than any of the existing interventions? |
| Baheiraei (2011) | Yes | Yes | Yes | No | No | Yes | Yes | Yes | Yes | Yes | Yes | Yes | No |
| Garcia (2022) | Yes | Yes | Yes | No | No | No | Yes | Yes | Yes | Yes | Yes | Yes | Can't tell |
| Rothstein (2022) | Yes | Yes | No | No | No | No | Yes | Yes | Yes | Yes | Yes | Yes | No |
| Sifunda (2019) | Yes | Yes | No | Yes | Yes | Yes | Yes | Yes | Yes | Yes | Yes | Yes | No |
| Cockcroft (2022) | Yes | Yes | Yes | Yes | Yes | Can't tell | Yes | Yes | Yes | Yes | Yes | Yes | Can't tell |

## Cross-sectional Studies

| First author’s last name (year) | 1. Were the criteria for inclusion in the sample clearly defined? | 2. Were the study subjects and the setting described in detail? | 3. Was the exposure measured in a valid and reliable way? | 4. Were objective, standard criteria used for measurement of the condition? | 5. Were confounding factors identified? | 6. Were strategies to deal with confounding factors stated? | 7. Were the outcomes measured in a valid and reliable way? | 8. Was appropriate statistical analysis used? |
| --- | --- | --- | --- | --- | --- | --- | --- | --- |
| Nasreen (2012) | Yes | Yes | Yes | Not applicable | Yes | No | Yes | Yes |
| Broadbent (2022) | Yes | Yes | Yes | Yes | Yes | Yes | Yes | Yes |
| Gavi | Not applicable | Yes | Unclear | Yes | Unclear | Unclear | Yes | Unclear |

## Quasi-experimental Studies

| First author’s last name (year) | 1. Is it clear in the study what is the ‘cause’ and what is the ‘effect’ (i.e. there is no confusion about which variable comes first)? | 2. Were the participants included in any comparisons similar? | 3. Were the participants included in any comparisons receiving similar treatment/care, other than the exposure or intervention of interest? | 4. Was there a control group? | 5. Were there multiple measurements of the outcome both pre and post the intervention/exposure? | 6. Was follow up complete and if not, were differences between groups in terms of their follow up adequately described and analyzed? | 7. Were the outcomes of participants included in any comparisons measured in the same way? | 8. Were outcomes measured in a reliable way? | 9. Was appropriate statistical analysis used? |
| --- | --- | --- | --- | --- | --- | --- | --- | --- | --- |
| Rahimi (2022) | Yes | Yes | Unclear | Yes | Yes | Yes | Yes | Yes | Yes |
| Su (2016) | Yes | Yes | Yes | Yes | Yes | Yes | Yes | Yes | Yes |
| Bich (2016) | Yes | Yes | Unclear | Yes | No | Yes | Yes | Yes | Yes |
| Jones (2008) | Yes | Yes | Yes | Yes | Yes | Yes | Yes | Yes | Yes |
| Lyatuu (2018) | Yes | Unclear | Unclear | Yes | Yes | Unclear | Yes | Yes | Unclear |
